# Letters to the Editor

**Published:** 1986-04

**Authors:** Cameron Kennedy, May Hobby


*Sir,*
*I have recently been looking after a delightful elderly lady who has expressed a number of pertinent thoughts about her medical management in 'poetry'. Do you think they would be of any interest for the Med-Chi Journal?*
*Yours etc*

**Cameron Kennedy**

To Dr. Kennedy, Dr. Grattan and all others concerned
From a miserable patient whose ulcer you've burned
In anticipation of an eventual cure
When no more of your torture shall I have to endure.
(The enclosed should have been written another few weeks hence
For it to make the least bit of sense).

Dear Doctors, thank you for all that you've done
I've had all the torture, you've had the fun
But now that I'm feeling quite fit and well
No amount of words my gratitude can tell.
To thank you all for your dedication
I am enclosing a small donation.
On research I would like it to be spent,
You will all know where it should be sent.

My stay here has been so very pleasant
But I've had enough of it for the present.
You take samples of this and then swabs of that
And then drain our blood like a vampire, or perhaps a small bat.
One day Dr. Grattan stood by my bed
'Dr. Kennedy suggested giving you Stromba', he said.
'Oh no, he didn't' I quickly replied
'The use of Stromba was never even implied'.
Dr. Grattan said 'Perhaps he called it such and such'.
'Yes', I agreed, 'but I don't know Double Dutch'.
I said 'Stromba always gives me cramp in the night,
If I pace the ward it will give other patients a fright'.

You discuss us in words that to us make no sense
Then walk away pleased at leaving us tense.
We wonder what further torment you have in store
When you have gone we moan that we can't take any more.
If ever I have to come here again
I hope you will use something that causes less pain.
With Eusol and paraffin, Varidase and Polyfax
I never felt comfortable enough to relax.
If there is a next time, to give me some heart,
Will you please use Jelaflex right from the start.
Then Dr. Grattan won't have to guess
With what awful substance my leg you dress
Or perhaps something better may by then be invented
That I hope will be sweeter than Varidase-scented.

Thank you again for healing my leg
If I've been a bad patient your pardon I beg.
I've not been put in the bathroom pit where, I understand
They shove all the patients who get out of hand
They then get flushed into the underground River Frome
And thus for yet more patients it makes some room.
Sister strongly denies this, she says 'Have no fear
There isn't the need for it because the mortuary is near'.

I don't know where you got the idea I'm a poet
I told you I write rubbish, well now you know it.
I've rather enjoyed my long stay in here
And in thanking you all I am really sincere.
I've noticed an appeal poster for research funds in the lobby
The enclosed is given to it by a grateful

